# Focused Teaching Improves Medical Student Professionalism and Data Gathering Skills in the Emergency Department

**DOI:** 10.7759/cureus.5765

**Published:** 2019-09-25

**Authors:** Colleen Smith, Antonios Likourezos, Joshua Schiller

**Affiliations:** 1 Assistant Professor Emergency Medicine, Medical Education and Simulation, Elmhurst Hospital, Icahn School of Medicine at Mount Sinai, New York, USA; 2 Emergency Medicine, Maimonides Medical Center, Brooklyn, USA

**Keywords:** resident as teacher, medical education, simulation, osce, emergency medicine, clerkship, communication, professionalism, data gathering, core competencies

## Abstract

Introduction: Leaders in medical education have developed milestones and core competencies in an attempt to ensure that relational skills, such as communication and professionalism, are emphasized in addition to the usual skills of medical knowledge, data gathering, and emergency stabilization during students' emergency medicine (EM) medical education. Providers facile in each of these areas have better patient outcomes, patient experiences, and decreased incidence of malpractice cases. The authors attempted to demonstrate that by deliberate teaching of these skills during an EM medical student clerkship, students could significantly improve their clinical performance.

Methods: This prospective, randomized, single-blinded cohort study was performed at an academic, tertiary, urban ED to investigate the effects of a one-on-one preceptor shift on the clinical performance of fourth-year medical students. Students were randomized into two groups and assessed by pre- and post-intervention objective structured clinical encounters (OSCEs) with standardized patients (SPs) at weeks one and three. A crossover design was employed so that students in the control group participated in a preceptor shift after their second OSCE. Measurements were based on a five-point Likert scale assessment linked to early EM milestones as defined by the Accreditation Council on Graduate Medical Education (ACGME).

Results: The mean improvement in total overall score was significantly greater in the intervention group: 4.31 versus 2.57 (Cohen’s d = 0.57, p = 0.029). When each milestone was assessed individually, students in the intervention group improved significantly in data gathering (Cohen’s d = 0.47, p = 0.048) and professionalism (Cohen’s d = 0.66, p = 0.011). There was a nonstatistically significant improvement for the intervention compared to control group in emergency management and communication skills. There was no improvement for either group in medical knowledge.

Conclusion: A one-on-one preceptor shift can result in a statistically significant improvement in data gathering and professionalism skills as measured by OSCEs.

## Introduction

Medical education in emergency medicine (EM) has largely been shaped by guidelines developed by the Association of American Medical Colleges (AAMC) and the Accreditation Council on Graduate Medical Education (ACGME), both of which have promoted a system of core competencies and milestones. The overall goal of these efforts is to shape the evaluation of students and residents in such a way that their training enforces skills and attributes necessary for successful daily practice and patient care [1-3]. These guidelines recognize the importance of not only of a strong background in medical knowledge, but also of a mastery of communication and professionalism skills necessary to provide compassionate, patient-centered care and to function in a diverse work environment. 

The benefit of competency-based learning is largely driven by evidence of improvements in patient safety and clinical outcomes, overall efficiency, professional and personal satisfaction, and in decreased medico-legal liability [4-7]. Interestingly, the incidence of lawsuits against EM physicians has decreased significantly since the introduction of competency-based learning [6-7]. Patient care has improved, as demonstrated by shorter lengths of stay in the ED and higher patient satisfaction [1, 7]. Additionally, residency training may be positively affected, with programs having the potential to demonstrate improved efficiency in accomplishing training objectives over a shorter time [8]. 

While medical schools commonly teach competency-based curricula to their students, the emphasis of teaching during the clerkship years often shifts away from a focus on relational skills, such as communication and professionalism, to mastery of clinical skills [2, 5-9]. In an attempt to incorporate all competencies essential to EM clinical care, our ED instituted a Resident as Teacher Preceptorship (RTP). This RTP program aimed to teach and enhance skills in five medical student competencies identified as vital in assessing clinical performance at this level of education: medical knowledge, data gathering, professionalism, communication skills, and emergency management [2-3]. We hypothesized that students who experienced the RTP intervention would show an improvement in all five competencies compared to those who received usual clerkship teaching alone.

## Materials and methods

Study design

This prospective, randomized, single-blinded cohort study was performed at an academic, tertiary, urban ED to investigate the effects of the RTP program on the clinical performance of fourth-year medical students during a four-week EM clinical clerkship from July 1, 2014 through March 31, 2015. The hospital’s Institutional Review Board approved the study prior to implementation.

A total of 65 students were randomized by coin flip into two groups prior to their arrival on site for a four-week clerkship. The intervention group consisted of 35 students while the control group had 30 students. There was no significant difference in this balance of students assigned to the two groups (p = 0.62).

**Figure 1:**
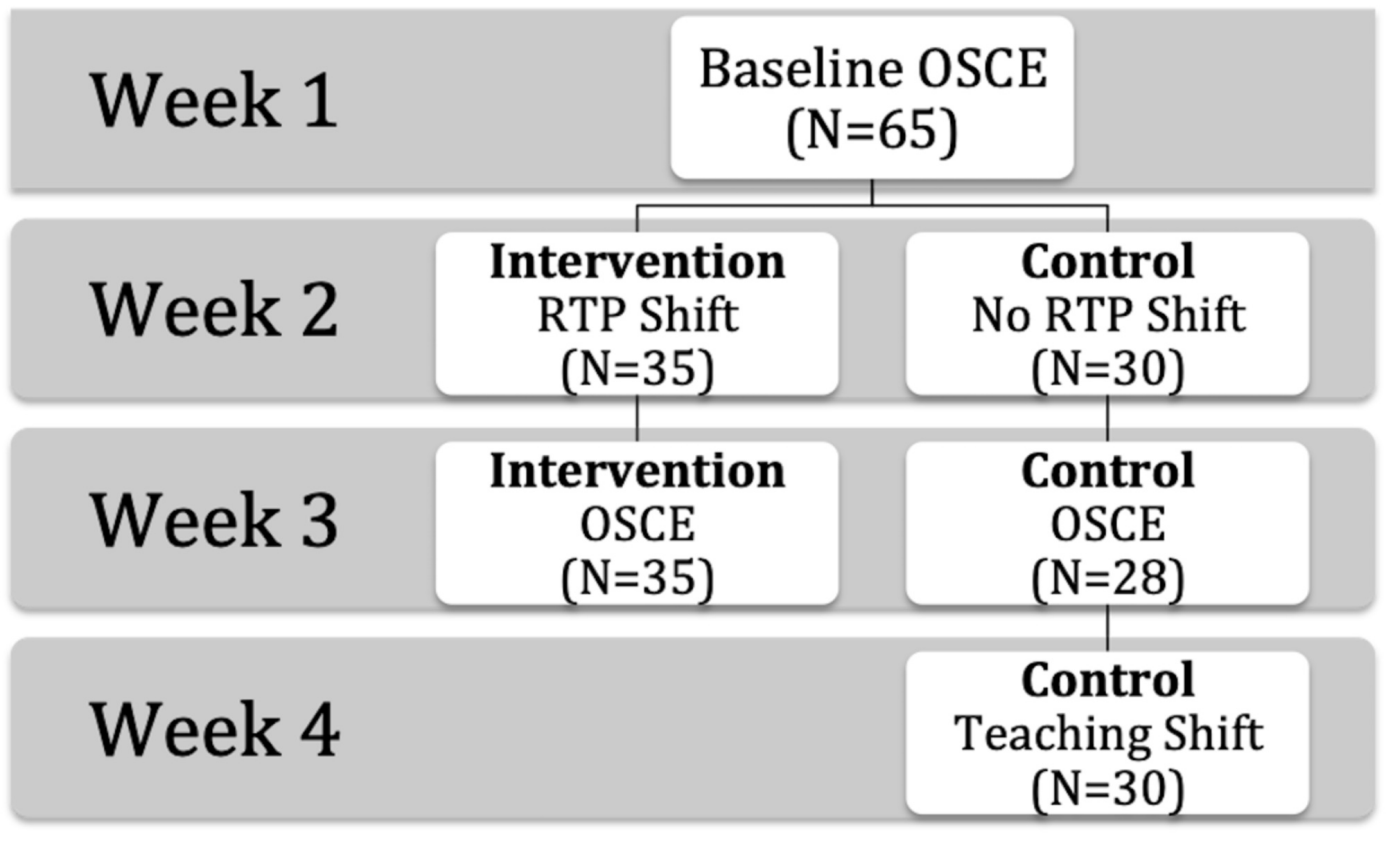
Study design. OSCE, objective structured clinical examination; RTP, resident as teacher preceptorship.

All medical students participated in an objective structured clinical examination (OSCE) with a standardized patient (SP). Each OSCE was specifically designed for this project to include a similar set of critical actions corresponding to the skills being assessed. They were then assigned to one-on-one RTP shifts in a crossover design either before or after a second OSCE for intervention and control groups, respectively (Figure 1). During these shifts residents and students saw patients at the students’ pace. Furthermore, the residents had no additional clinical responsibilities other than teaching and overseeing the medical students. Most students were able to see two to three patients per RTP shift. 

Residents who led the RTP shifts were all in their third and final year of residency. They were self-selected and had mentored and taught medical students intermittently throughout residency. In preparation for them to lead RTP shifts, they participated in a targeted two-hour teaching seminar that exposed them to specific topics of adult learning theory and organizational tools [5-6]. Resident teachers guided students through all aspects of patient care. They were specifically tasked with focusing on relational skills including issues of professionalism and specific communication tools for the variety of circumstances encountered in the ED [10].

Study population

Medical students participating in the study were in their fourth year and rotated to our ED from a variety of medical schools. The students’ ED clinical experience ranged from none to two previous rotations. A total of 92.3% (60/65) of these students applied to EM residencies during the academic year in which they rotated. 

Both the intervention and control group were comparable with regard to demographic characteristics and overall clerkship performance. The intervention group consisted of 22 males (63%) while the control group had 18 male students (64%); p = 0.91. The median overall clerkship grade for the intervention group was 85.1 (range: 77-92) and for control group it was 86.0 (range: 79-94); p = 0.67. In the intervention group, 32 students (91.4%) applied to EM residency while 26 of the controls (92.9%) applied to EM; p = 0.84. Two students in the control group were unable to take the second OSCE due to conflicts with residency interviews and were subsequently excluded from the data analysis.

Assessment

Students’ learning outcomes were evaluated based on median change in scores between the two different OSCEs. Both OSCE cases provided opportunities for medical students to: greet a patient, perform a history and physical examination, decide on escalating management interventions, explain the situation to the patient, present to a consultant, and hand off to an admitting physician. Each required action involves aspects of the ACGME competencies being measured. 

Scoring of each OSCE was done by the same blinded attending physician rater. Rating was based on a Likert scale from zero to five. The scale contained anchors derived directly from corresponding ACGME Milestones for Emergency Medicine, or from a combination of milestones, in each of the competencies: patient-centered communication (milestone 22), professional values (milestone 20), emergency management (milestones 1,3,4,7), data gathering (milestones 2,3,6), and medical knowledge (milestone 15) [2, 7].

Data analysis

Data were described in terms of frequency (%) for categorical variables, median and range for continuous variables with outliers, and mean ± standard deviation for normally distributed variables. Likewise, the chi-square test was used to compare group differences relative to categorical variables, the Mann-Whitney test compared group differences in terms of medians, and either t-tests or repeated measures analysis of variance were used for looking at group differences in terms of means or mean change. Differences in improvement were reported in terms of raw effect size and in terms of the standardized effect size or Cohen’s d. 

Cronbach’s alpha was used to determine the internal consistency of the anchors measuring the OSCE objectives being taught. This is typically considered an estimate of the reliability of the items; any alpha value less than 0.70 was considered to show inadequate reliability. All data analyses were done using IBM SPSS 23.0 (IBM Corp., Armonk, NY) and a level of significance < 0.05 was used for all statistical tests. Based on detecting at least a one point difference in rating between the groups on any one competency evaluation and assuming a standard deviation of 1.25 (estimated by dividing four into the range of possible ratings from 0 to 5) we calculated that we would need at least 26 students in each group for 80% power with alpha = 0.05.

## Results

As shown in Table 1, the mean improvement in total overall score was significantly greater in the intervention group: 4.31 versus 2.57 (Cohen’s d = 0.57, p = 0.029). We chose to calculate the overall score without medical knowledge because it was not the focus of the intervention. When broken down into each component milestone, this statistically significant improvement persisted in two areas: data gathering (Cohen’s d = 0.47, p = 0.048); and professionalism (Cohen’s d = 0.66, p = 0.011). 

**Table 1:** Mean change in OSCE scores for each group. OSCE, objective structured clinical examination. *Overall score = data gathering, emergency management, professionalism, communication.

Objective	Intervention (N=35)	Control (N=28)	Effect size (Cohen’s d)	p-value
Overall score*	4.31 ± 3.28	2.57 ± 2.78	1.74 (0.57)	0.029
Data gathering	0.97 ± 0.98	0.50 ± 0.84	0.47 (0.51)	0.048
Emergency management	1.20 ± 0.93	0.86 ± 1.01	0.34 (0.35)	0.167
Professionalism	1.14 ± 0.88	0.57 ± 0.84	0.57 (0.66)	0.011
Communication	1.00 ± 1.08	0.64 ± 0.91	0.36 (0.36)	0.169
Medical knowledge	0.60 ± 0.98	0.61 ± 0.99	-0.01 (-0.01)	0.98

Cohen’s d for the remaining areas included in the total score (emergency management and communication) were at least 0.35, or well above a small standardized difference. Medical knowledge showed no improvement between the groups (Cohen’s d = -0.01). Because medical knowledge was not the focus of the intervention and its measurement was complicated by comparing two different clinical OSCE scenarios, it was omitted from the total score calculations. The fact that overall the mean scores improved more in the intervention group, as we expected, supports the scoring rubric as having construct validity.

## Discussion

The results of our study supports a growing body of literature that focused teaching in relational skills, particularly in the areas of professionalism and data gathering, can result in a greater improvement in learner behavior compared to usual clerkship teaching, as measured by OSCE [6,11-15]. That this teaching is imperative is further supported by evidence that providers at any level perform uniformly poorly when randomly tested on relational skills without preceding educational interventions [16]. 

The greater improvement in data gathering is especially interesting. While generally considered to be a technical clinical skill, gathering precise and focused data relies heavily on communication ability. Our study demonstrates a nonsignificant positive effect of the RTP intervention on the broader category of communication skills. This suggests that improvement in relational competencies such as communication may lead to refinement of all competencies. 

Our study is unique in that it is one of a few studies in the body of resident as teacher literature that attempts to demonstrate a change in learner behavior after initiating an RTP [12,17-19]. The majority of resident as teacher studies have focused on improving residents’ teaching skills through direct observation and surveying medical students’ perception of their own learning [19-20]. Additionally, rather than a focus on a mastery of a single procedure or skill, our study attempts to measure impact of the RTP in both traditional clerkship areas (such as medical knowledge, data gathering, and emergency management), as well as in the broader array of relational skills (namely professionalism and communication) necessary to succeed in the clinical work environment in which we operate today. 

Historically, it has been difficult to objectively assess relational skills without implicit observer bias [21]. Thus, appropriate measure of the effects of training for these skills remains elusive. In fact, many resident physicians’ perceptions of such skills are highly variable [22]. As such, the single observer format along of this study may circumvent potential pitfalls in assessment of learner skills. 

In this same vein, there is some question as to the use of OSCE as an assessment tool to evaluate learner performance, as Hill et al. mention in one similar study [17, 23]. Evaluation by OSCE is known to be subject to the Hawthorne effect, a well-described phenomenon whereby individuals improve or modify their usual behavior based on their awareness of being observed [24]. The authors posit that comparison of students to themselves through pre- and post-intervention OSCE negates some of the impact of this effect. While the question remains whether performance during an OSCE is indicative of either learning or future behavior of a clinician in practice, the OSCE is increasingly utilized as an assessment tool [23-25].

Our study suggests several areas for further research. The long-term impact of this particular intervention on learners as they transition into residency should be assessed. Furthermore, considering data from simulation literature that educational effects of simulation interventions deteriorate over time, a next step for this study would be implementation and assessment of outcomes of a longitudinal curriculum focused on relational skills throughout clerkship years [26]. Finally, other modalities of assessment could be incorporated into measurements of learner outcomes such as comparison to United States Medical Licensing Examination scores and the introduction of unannounced or in-situ SPs and video encounters to help obviate the Hawthorn effect.

Limitations

As with most single institution studies, sample size is a considerable limitation. However, we were able to enroll more students than needed based on our original power calculations. The loss of two students from the control group could have impacted the results either positively or negatively because of the small overall sample size. A second limitation is the fact that only one blinded rater evaluated students in all OSCEs. Finally, the pre- and post-intervention OSCEs were different cases, which could result in some variability in performance. However, had both OSCE cases been the same we would have expected improvement simply because of familiarity with the case.

## Conclusions

Medical students who received the RTP intervention demonstrated a short-term improvement in the core EM competencies of data gathering and professionalism as compared to medical students who receive typical clerkship teaching alone. This suggests that providing resident teachers with didactic and clinical tools for teaching students within the ACGME framework of competencies could result in improved learner performance in relational skills.

## Appendices

Rating scale for each competency 

0 = None to few anchors effectively performed, to 5 = Most to all anchors effectively performed

Anchors for each competency

Data Gathering/Synthesis

Communicates a reliable history. Identifies past medical history, medications, and allergies.  Performs a focused physical exam. Constructs a list of potential diagnoses based on chief complaint and initial assessment.

Performs and communicates a focused history and physical exam which effectively addresses the chief complaint and urgent patient issues. Constructs a list of potential diagnoses with the greatest potential for morbidity or mortality, as well as based on the greatest likelihood of occurrence.

Elicits essential components of a history given multiple possibilities on diagnoses. Covers questions about multiple systems that would address cardiovascular, pulmonary, infectious, gastrointestinal, etc. etiologies. Uses information from history and physical to develop ranked differential including those with the greatest potential for morbidity or mortality. Correctly identifies “sick versus not sick” patients.

Synthesizes essential data necessary for the correct management of patients using all potential sources of data. Prioritizes information to narrow the list of weighted differential diagnoses in determining appropriate management. 

Considers multiple processes to provide complete care. Avoids premature closure. Identifies obscure, occult, or rare patient conditions based solely on historical and physical exam findings. 

Emergency Care

Prioritizes critical initial stabilization action and mobilizes hospital support services in the resuscitation of a critically ill or injured patient and reassesses after stabilizing intervention.

Recognizes abnormal vital signs.

Recognizes when a patient is unstable requiring immediate intervention. Provides oxygen support, establishes IV access, and puts patient on monitor.

Emphasizes initial stabilization actions, over other actions, in the resuscitation of a critically ill patient. Will ensure that airway/respiratory status is stabilized before moving on to other assessments. 

Reassesses after implementing a stabilizing intervention to ascertain further management.  Recognize that patient is not stable for leaving ED.

Integrates consulting services into a management strategy for continued stabilization. Will obtain appropriate disposition to ensure monitoring and care needs met (i.e. choose appropriate level of care - step down, intensive care unit (ICU)).  

Professionalism

Demonstrates compassion, integrity, and respect for others as well as adherence to the ethical principles relevant to the practice of medicine.

Demonstrates behavior that conveys caring, honesty, genuine interest, and tolerance.  Responds to patient’s anxieties effectively by offering empathetic attitude during management.

Demonstrates an understanding of the importance of compassion, integrity, respect, sensitivity, and responsiveness and exhibits these attitudes during the case.  Takes moments to calm patient throughout the case. 

Incorporates importance of recognizing patient’s personal beliefs and values in patient’s medical care.  Keeps patient informed as to each step of medical management. 

Develops an appropriate approach to prioritizing patient’s best interest in all relationships and situations; manages patient concerns in order to facilitate care.  Will fully describe the need for interventions to patient and address concerns as appropriate.

Demonstrates organizational strategies to maintain patient interests throughout management to disposition.  Shows respect for patient aversion to be admitted; will successfully show understanding for patient anxieties while also acting in patient’s best interest, ultimately rendering disposition with full patient support.

Patient-Centered Communication

Demonstrates interpersonal and communication skills that result in the effective exchange of information and collaboration with patients and their families.

Establishes rapports with patient, and demonstrates empathy.  Listens effectively to patient complaints.  Introduces him/herself.  Maintains professional level of communication.

Elicits patients’ reasons for seeking medical care, and expectations from the ED visit.  Able to soothe anxieties associated with patient complaints.  Explains interventions before performing them. 

Manages the expectations of those who receive care in the ED and uses communication methods that minimize the potential for stress, conflict, and misunderstanding.  Conveys warmth and reassurance during workup.

Uses flexible communication strategies and adjusts them based on the clinical situation to resolve specific ED challenges.  Elicited by prompt by patient that he is getting highly upset at his condition. 

Presents the patient completely and systematically when signing out to the consultant (ICU fellow), providing a full history and physical with assessment and plan for admission and justifications for workup, interventions, and medications given.

Medical Knowledge

Demonstrates appropriate medical knowledge in the care of emergency medicine patients.

Asthma case: Recognizes acute signs of respiratory or circulatory distress.

Considers back up therapies for refractory reactive airway.  Considers epinephrine and magnesium.

Establishes the possibility of chronic obstructive pulmonary disease (COPD). Orders imaging and appropriate antibiotics to address a possible underlying pulmonary infection.

Dispositions patient in appropriate manner (ICU), given unstable respiratory status and need for continuous nebulization.

Abdominal aortic aneurysm case:  Recognizes acute signs of hemodynamic compromise (i.e. hypotension, weakness, and dizziness).

Identifies cardiovascular compromise as initial problem that needs to be addressed.  Considers differential diagnosis of shock to determine most likely etiology (hypovolemia). 

Considers back up therapies for refractory hypotension (emergent transfusion). Considers appropriate imaging-abdominal series X-rays for perforation/widened mediastinum, abdominal ultrasound. 

Recognizes why HR was not tachycardic (beta-blockade).

Able to properly interpret labs and imaging to recognize acute ruptured aortic aneurysm.  Suggest massive transfusion protocol as needed or at least consider platelet transfusion as patient is on aspirin and clopidogrel. 

Dispositions patient in appropriate manner, (vascular or general surgery to operating room), given unstable cardiovascular status and need for surgical repair.

## References

[1] Institute of Medicine (US) Committee on the Health Professions Education Summit (2003). Chapter 3. The core competencies needed for health care professionals. Health Professions Education: A Bridge to Quality.

[2] Santen SA, Peterson WJ, Khandelwal S, House JB, Manthey DE, Sozener CB (2014). Medical student milestones in emergency medicine. Acad Emer Med.

[3] (2019). Accreditation Council for Graduate Medical Education. Program requirements for graduate medical education in emergency medicine. https://www.acgme.org/Portals/0/PFAssets/ProgramRequirements/CPRResidency2019.pdf.

[4] Carney PA, Palmer RT, Fuqua Miller M (2016). Tools to assess behavioral and social science competencies in medical education: a systematic review. Acad Med.

[5] Mauksch L, Farber S, Greer HT (2013). Design, dissemination, and evaluation of an advanced communication elective at seven U.S. medical schools. Acad Med.

[6] Smith S, Hanson JL, Tewksbury LR, Christy C, Talib NJ, Harris MA, Wolf FM (2007). Teaching patient communication skills to medical students: a review of randomized controlled trials. Eval Health Prof.

[7] Cherney AR, Smith AB, Worrilow CC (2018). Emergency medicine resident self-assessment of clinical teaching compared to student evaluation using a previously validated rubric. Clin Ther.

[8] van Rossum TR, Scheele F, Sluiter HE, Bosman PJ, Rijksen L, Heyligers IC (2018). Flexible competency based medical education: more time efficient, higher costs. Med Teach.

[9] Santen SA, Rademacher N, Heron SL, Khandelwal S, Hauff S, Hopson L (2013). How competent are emergency medicine interns for level 1 milestones: who is responsible?. Acad Emer Med.

[10] Ruest A, Smith C, Gagdil R (2015). Resident-based preceptorship improves student clinical skills in the emergency department. West JEM.

[11] Blackmore A, Kasfiki EV, Purva M (2018). Simulation-based education to improve communication skills: a systematic review and identification of current best practice. BMJ STEL.

[12] Hill AG, Srinivasa S, Hawken SJ, Barrow M, Farrell SE, Hattie J, Yu TC (2012). Impact of a resident-as-teacher workshop on teaching behavior of interns and learning outcomes of medical students. J Grad Med Educ.

[13] Schmitz FM, Schnabel KP, Stricker D, Fischer MR, Guttormsen S (2017). Learning communication from erroneous video-based examples: a double-blind randomized controlled trial. Pat Educ Coun.

[14] Maatouk-Bürmann B, Ringel N, Spang J (2016). Improving patient-centered communication: results of a randomized controlled trial. Pat Educ Coun.

[15] Dong T, LaRochelle JS, Durning SJ, Saguil A, Swygert K, Artino AR (2015). Longitudinal effects of medical students' communication skills on future performance. Mil Med.

[16] Wouda JC, van de Wiel HBM (2012). The communication competency of medical students, residents and consultants. Pat Educ Coun.

[17] Hill AG, Yu TC, Barrow M, Hattie J (2009). A systematic review of resident-as-teacher programs. Med Educ.

[18] Ahn J, Jones D, Yarris LM, Fromme HB (2017). A national needs assessment of emergency medicine resident-as-teacher curricula. Int Emer Med.

[19] Krzyzaniak SM, Cherney A, Messman A (2018). Curated collections for educators: five key papers about residents as teachers curriculum development. Cureus.

[20] Chokshi BD, Schumacher HK, Reese K (2017). A "resident-as-teacher" curriculum using a flipped classroom approach: can a model designed for efficiency also be effective?. Acad Med.

[21] Schnapp BH, Ritter D, Kraut AS, Fallon S, Westergaard MC (2019). Assessing residency applicants' communication and professionalism: standardized video interview scores compared to faculty gestalt. West JEM.

[22] Cho CS, Delgado EM, Barg FK, Posner JC (2013). Resident perspectives on professionalism lack common consensus. Ann Emerg Med.

[23] Bodamer C, Feldman M, Kushinka J, Brock E, Dow A, Evans JA, Bearman G (2015). An internal medicine simulated practical examination for assessment of clinical competency in third-year medical students. Simul Healthc.

[24] McCambridge J, Witton J, Elbourne DR (2014). Systematic review of the Hawthorne effect: new concepts are needed to study research participation effects. J Clin Epid.

[25] Casey PM, Goepfert AR, Espey EL (2009). To the point: reviews in medical education--the objective structured clinical examination. Am J Ob Gyn.

[26] Steinemann S, Berg G, Skinner A (2011). In situ, multidisciplinary, simulation-based teamwork training improves early trauma care. J Surg Educ.

